# Evaluation of the Expression and Localization of the Multifunctional Protein CacyBP/SIP and Elements of the MAPK Signaling Pathway in the Adrenal Glands of Rats with Primary and Secondary Hypertension

**DOI:** 10.3390/ijms25010084

**Published:** 2023-12-20

**Authors:** Magdalena Smereczańska, Natalia Domian, Maryla Młynarczyk, Anna Pędzińska-Betiuk, Irena Kasacka

**Affiliations:** 1 Department of Histology and Cytophysiology, Medical University of Bialystok, 15-222 Bialystok, Poland; 2 Department of Experimental Physiology and Pathophysiology, Medical University of Bialystok, 15-222 Białystok, Poland

**Keywords:** CacyBP/SIP, p-ERK1/2, p-p38, hypertension, adrenal glands

## Abstract

Hypertension is a global civilization disease and one of the most common causes of death in the world. Organ dysfunction is a serious health consequence of hypertension, which involves damage to the heart, kidneys and adrenals. The interaction of recently discovered multifunctional protein-CacyBP/SIP with ERK1/2 and p38 kinases by regulating the activity and intracellular localization of these kinases may play an important role in the signaling pathways involved in the pathogenesis of hypertension. Due to the lack of data on this subject, we decided to investigate the localization, expression and possible relationship between the studied parameters in the adrenals under arterial hypertension. The study was conducted on the adrenals of rats with spontaneous and DOCA-salt hypertension. The expression of CacyBP/SIP, p-ERK1/2 and p-p38 was detected by immunohistochemistry and qRT-PCR. The results show a statistically significant decrease in CacyBP/SIP expression in the adrenal glands of hypertensive rats. With ERK1/2, there was a decrease in cortical immunoreactivity and an increase in the adrenal medulla of primary hypertensive rats. In contrast, in the adrenals of DOCA-salt rats, ERK1/2 immunoreactivity increased in the cortex and decreased in the medulla. In turn, p38 expression was higher in the adrenal glands of rats with primary and secondary hypertension. The obtained results may suggest the involvement of CacyBP/SIP in the regulation of signaling pathways in which MAP kinases play an important role and provide new insight into molecular events in hypertension. Moreover, they show the participation of CacyBP/SIP in response to oxidative stress.

## 1. Introduction

Hypertension is a global disease of civilization and one of the most common causes of death in the world. Since hypertension is characterized by a latent and insidious course, the disease very often remains undiagnosed. Organ dysfunction is a serious health consequence of hypertension. The risk of damage especially affects the heart, kidneys, brain and adrenal glands. Organs such as the heart and brain have been well studied in animal models of hypertension. However, the mechanisms accompanying the proven disturbances in the secretion of both mineralocorticoids and catecholamines in hypertension have not been fully elucidated.

There is a close relationship between the proper functioning of the adrenal glands and hypertension. Aldosterone, produced by the adrenal glands, plays an important role in maintaining blood pressure homeostasis. Persistently elevated blood pressure may also be the result of the increased secretion of cortisol by the cells of the zona fasciculata or abnormal metabolism of this hormone. In turn, catecholamines released from the adrenal medulla are involved in the neurohormonal regulation of blood pressure, and their relationship with hypertension is well documented. The dysregulated activity of adrenal medullary cells may be caused by oxidative stress accompanying hypertension, which leads to the production of inflammatory mediators. Numerous literature reports indicate that hypertension often results from overactivity of the sympathetic nervous system and disturbed balance in the RAA system [1,2,3]. Moreover, it has been shown that the MAPK signaling cascade is involved in the regulation of steroidogenesis by influencing the expression of the steroidogenesis acute regulatory protein—StAR, which facilitates the rapid movement of cholesterol from the outside to the inner mitochondrial membrane, which enables de novo biosynthesis of steroid hormones in response to an increase in ACTH or angiotensin II. Literature data indicate that angiotensin II-induced activation of MAP kinases is responsible for aldosterone secretion by increasing the expression of specific steroidogenic proteins, such as StAR [4,5]. 

The cause of hypertension is identified in approximately 10% of cases (secondary hypertension), while in 90% of cases the etiology is not determined (primary hypertension).

CacyBP/SIP (calcyclin binding protein/Siah-1interacting protein) is a multifunctional protein that is involved in many important cellular processes. Higher levels of CacyBP/SIP expression are noted in the brain and spleen, moderate levels in the heart muscle and liver, and low levels in the kidneys and lymph nodes. Increased levels of CacyBP/SIP were also observed in the nuclei of cancer cells [6]. It can influence the processes of cell proliferation and differentiation, reorganization of the cytoskeleton, ubiquitination and stress response. Despite numerous studies, the role of CacyBP/SIP in the discussed processes has not been fully explained [7,8]. Studies to date have shown that CacyBP/SIP interacts with several different proteins. These include proteins from the S100 family, components of the E3 ubiquitin ligase complex: Skp1 and Siah-1, Hsp90 protein, cytoskeleton proteins: actin, tropomyosin, tubulin, tau protein, and MAP family kinases: ERK1/2 and p38 [8].

Recent literature data suggest that the role of CacyBP/SIP in various signaling pathways may be related to the dephosphorylation of MAP kinases. MAPKs regulate the activity of enzymes and transcription factors. The range of biological activity of these kinases is very wide, which indicates their participation in the pathogenesis of many diseases, including hypertension [7,9]). These kinases have been shown to be involved in the activation of inflammatory processes and the recruitment of macrophages and monocytes, smooth muscle cell proliferation and endothelial cell activity [10,11]. 

MAP kinases regulate the response of cells to changes in environmental conditions, e.g., their adaptation to ischemia or hypoxia [12]. ERK1/2 activation has been suggested to contribute to vascular smooth muscle cell hypertrophy and hyperplasia, leading to an increase in peripheral resistance and an increase in blood pressure [13]. This is related to the higher expression of growth factors and cytokines, which stimulate MAPK by binding to appropriate receptors. This results in the transmission of a signal that activates myocytes. The phosphatase protein has been shown to downregulate mitogen-activated kinases. Lower phosphatase expression associated with reduced ERK1 dephosphorylation may contribute to myocyte proliferation in the injured vessel [13].

It has been shown that p38 kinase may be involved in maintaining tissue homeostasis and in some pathological conditions, including cardiovascular diseases, cancer and inflammatory processes. Numerous reports indicate that p38 kinase is activated by a number of pro-inflammatory cytokines, and its activation results in the recruitment and release of additional pro-inflammatory cytokines, mostly IL-1 and TNFα, IL-8 in response to IL-1 or osmotic shock and IL-6 in response to TNFα [14]. On this basis, p38 is believed to play a key role in numerous signaling pathways involved in initiating and maintaining inflammation (chronic inflammation) in many pathological states [15]. P38 mediates attenuation of nitric oxide-dependent vasodilatory function of the endothelium. By activating p38, C-reactive protein (CRP) has also been shown to inhibit this endothelial function [16]. The pathogenesis of hypertension is still not fully understood, which is why new mechanisms are still being discovered that may be important in the treatment of hypertension. The interaction of CacyBP/SIP with ERK1/2 and p38 by regulating the activity and intracellular localization of these kinases, perhaps play an important role in the signaling pathways related to the sympathetic nervous system or the renin angiotensin aldosterone system, which are involved in the pathogenesis of hypertension. Due to the lack of data on this subject, we decided to investigate the localization, expression and possible relationship between the studied parameters in adrenal glands in experimental hypertension. These are preliminary pilot studies aimed at providing information on the level of expression of the studied parameters and the possible correlation between CacyBP/SIP and MAP kinases, which will be the basis for further, more detailed studies of functional interaction of these proteins in hypertension of various etiology. 

The results of our research may provide new information on the development of various forms of hypertension, which allows us to hope for the development of therapeutic protocols based on biochemical and cytophysiological data in the future. Finding new, potentially important targets for antihypertensive drugs that develop their action through targeted interactions with regulatory pathways based on CacyBP/SIP/MAP kinase signaling cascades seems worth further research.

The aim of the study is the comparative assessment of the expression of CacyBP/SIP, p-ERK1/2 and p-p38 in the adrenal glands of rats with primary and secondary hypertension.

## 2. Results

Systolic hypertension was found in all rats of the study groups (SHR and DOCA-salt). Mean BP values for each of the normotensive and hypertensive groups are presented in Table 1. 

**Figure 1:**
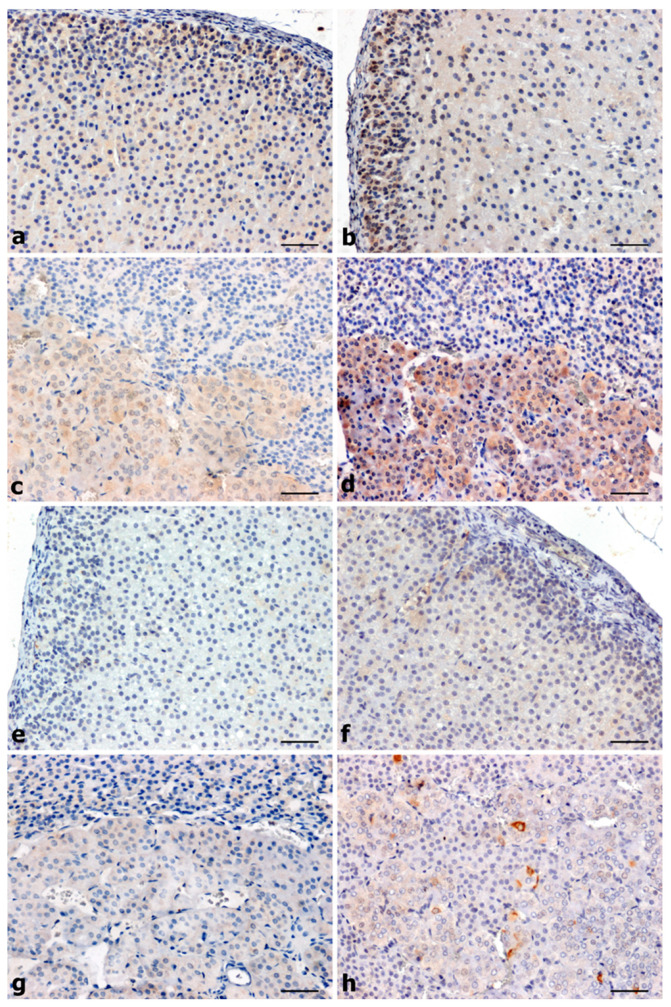
Adrenal distribution of CacyBP/SIP determined by immunohistochemistry: (**a**–**d**) normotensive rats, (**e**–**h**) hypertensive rats. Positive immunostaining was present in the cytoplasm of endocrine cells in the glomerular layer and medulla of WKY animals with moderate intensity (**a,c**), and a much stronger reaction was present in UNX (**b**,**d**). Negative in cortex and very weak in the medulla of SHR (**e**,**g**) and significantly attenuated in whole organ, relative to UNX in DOCA-salt (**f**,**h**). Scale bars: 50 μm.

Immunohistochemical reaction showing CacyBP/SIP was positive in both adrenal cortex and medulla of control group rats (Figure 1a–d). The intensity of the reaction in the adrenal glands of UNX animals was the strongest, both in the cortex (glomerular layer) and in the adrenal medulla compared to all tested rats (Figure 1b,d).

In SHR, CacyBP/SIP-immunoreactivity was negative in the cortex and trace in the medullary part of the organ (Figure 1e,g). A significant attenuation-specific signal for CacyBP/SIP to UNX was observed in the adrenal glands of DOCA animals (Figure 1f,h).

The localization of CacyBP/SIP in the adrenal glands of rats with primary and secondary hypertension showed the presence of this protein mainly in the cytoplasmic compartment of the cortex and medulla (Figure 1e–h).

**Figure 2:**
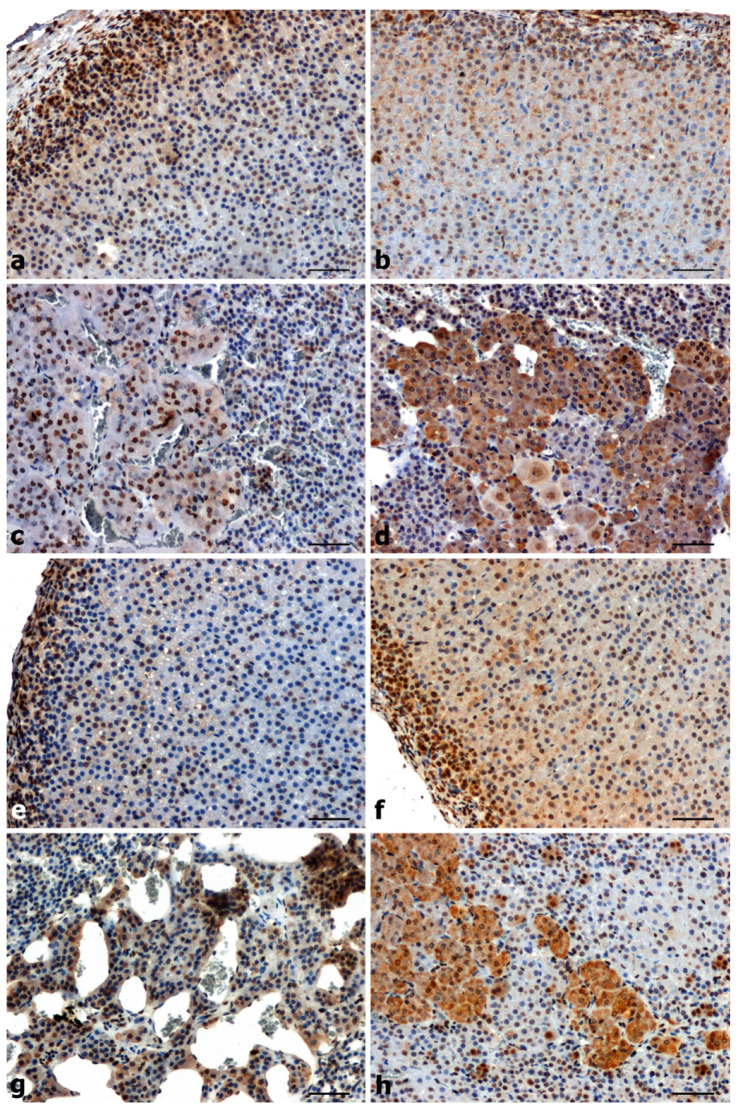
Immunoreactivity of p-ERK1/2 in the adrenal glands of normotensive (**a**–**d**) and hypertensive (**e**–**h**) rats. Strong positive immunostaining in cytoplasmic location of glomerular cells (**a**) and moderate in adrenal medulla of WKY (**c**), positive reaction of moderate intensity in the cytoplasm of the cells of the glomerular and fasciculata layers (**b)** and strong in the medulla (**d**), positive immunosignal in the cortex (**e**) and strong in the medulla (**g**) in SHR, higher expression in the cortex (**f**) and lower in the medulla (**h**) of DOCA-salt. Scale bars: 50 μm.

A positive reaction with the anti-p-ERK1/2 antibody was found in the adrenal glands of all rats (Figure 2a–h). p-ERK1/2 immunoreactivity was strongly positive in the glomerular layer and weak in the zona fasciculata and in the medulla of adrenals of WKY rats (Figure 2a,c). In UNX control rats, a moderate intensity reaction was observed in the cytoplasm of glomerular and fasciculata cells and a very strong p-ERK1/2 staining in the adrenal medulla (Figure 2b,d). p-ERK1/2 immunoexpression in the SHR adrenal cortex is reduced (Figure 2e), while in the medulla it is increased (Figure 2g) compared to control WKY (Figure 2a,c). In the adrenals of DOCA-salt hypertensive rats, there was an increase in p-ERK1/2 immunoreactivity in the cortex and a decrease in the medulla (Figure 2f,h) compared to UNX control rats (Figure 2b,d).

The localization of p-ERK1/2 in the adrenal glands of rats with primary and secondary hypertension showed the presence of this kinase mainly in the nuclear and cytoplasmic localization of the glomerular layer of the cortex and in the cytoplasmic compartment of the medulla (Figure 2e–h).

**Figure 3:**
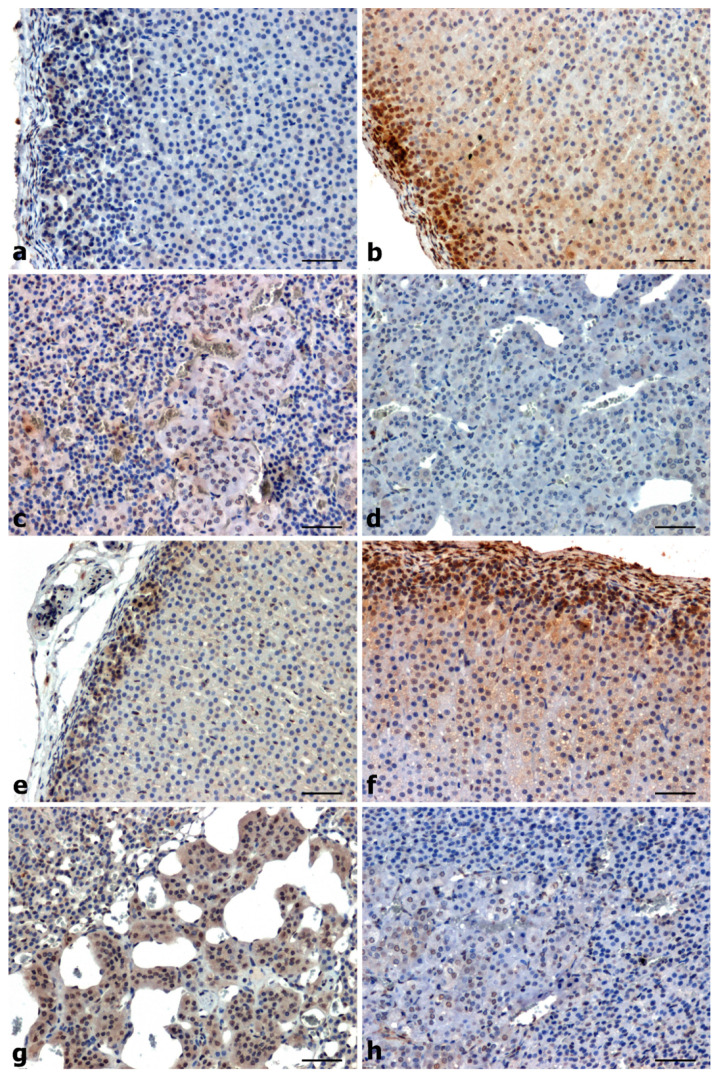
Immunohistochemical staining to demonstrate localization of p-p38 in the adrenal glands of normotensive (**a**–**d**) and hypertensive (**e**–**h**) rats. WKY—very weak signal in the cytoplasm of cells of the glomerular layer (**a**) and moderate in the medulla (**c**). UNX—strong reaction in the glomerular layer and moderate in the zona fasciculata (**b**) and very weak in the medulla (**d**). SHR—increased reaction in the cytoplasm of the glomerular layer (**e**) and in the medulla (**g**), DOCA-salt—increased reaction in the glomerular layer and in the zona fasciculata (**f**) and very weak signal in the medulla (**h**). Scale bars: 50 μm.

In SHR, an increase in p-p38 immunoreactivity was observed in the glomerular layer of the adrenal cortex (Figure 3e) and in the adrenal medulla (Figure 3g) compared to WKY normotensive rats (Figure 3a,c). The intensity of p-p38 immunostaining in rats with secondary DOCA-hypertension was very strong in the glomerular layer of the adrenal cortex (Figure 3f) and stronger compared to UNX animals (Figure 3b), while in the adrenal medulla the specific signal was very weak (Figure 3h) and comparable to that observed in UNX control animals (Figure 3d).

The localization of p-p38 in the adrenal glands of hypertensive animals was characteristic mainly of the glomerular layer of the cortex, both for the cytoplasmic compartment and nuclear localization, and for the cytoplasmic localization of the medulla in primary hypertension (Figure 3e–g).

Image morphometric analysis results are presented in Table 2.

Due to the lack of statistically significant correlation between the tested proteins in primary and secondary hypertension (*p* > 0.05), the results of this analysis are included in the tables as Supplementary Materials (Tables S1 and S2).

**Figure 4:**
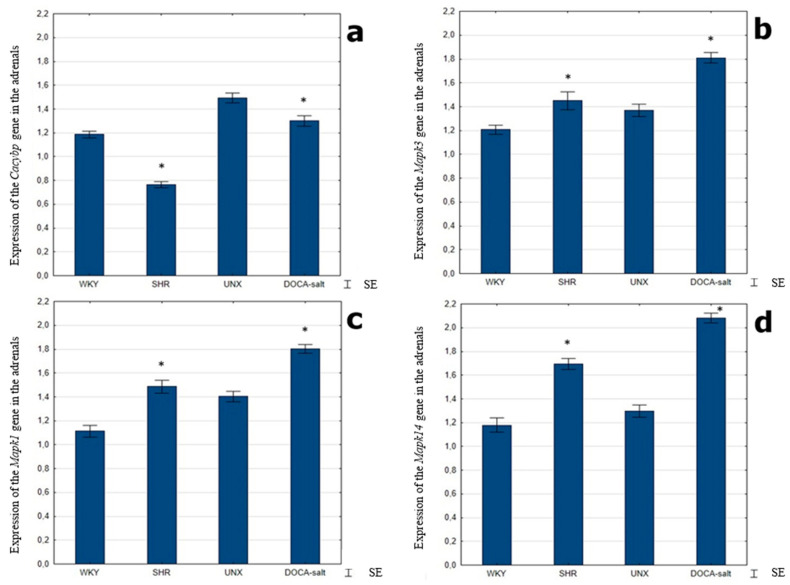
Expression of the *Cacybp*, *Mapk3*, *Mapk1* and *Mapk14* genes in the adrenal glands of control and hypertensive rats (**a**–**d**), * *p* < 0.05—hypertension vs. control, SE—standard error.

RT-qPCR analysis revealed lower expression of the CacyBP/SIP gene in the adrenal glands of all hypertensive animals compared to the corresponding control group (Figure 4a). For the *Mapk3* and *Mapk1* gene, significantly higher expression, with a similar profile for both genes, was detected in the adrenal glands of hypertensive rats compared to the corresponding normotensive rats (Figure 4b,c). Detailed analysis of the RT-qPCR results also revealed a significant higher expression of the gene encoding p38 in the adrenals of hypertensive rats compared to the corresponding control group (Figure 4d).

## 3. Discussion

Cardiovascular diseases, including hypertension, are one of the leading causes of premature death. Therefore, the efforts of clinicians and scientists to obtain better and more effective results of the pharmacological strategy continue. Due to the complex, multifactorial mechanism of the physiological regulation of blood pressure involving many signaling pathways, the pathophysiology of hypertension is still not fully understood [2,3]. 

In search of new mechanisms involved in the pathogenesis of hypertension, this research is a pilot study and an introduction to the assessment of a certain functional relationship of CacyBP/SIP with ERK1/2 and p38 kinases in the adrenal glands of hypertensive rats, which may contribute to broadening our knowledge on the interaction of CacyBP/SIP protein with MAP kinases.

To the best of our knowledge, this study is the first to investigate the localization and expression of CacyBP/SIP, ERK1/2 and p38 in the adrenal glands in the setting of hypertensive of various etiologies.

The analysis of the obtained results showed lower expression of the gene encoding CacyBP/SIP in rats with spontaneous and secondary hypertension. The results of studies conducted so far have shown the significant role of CacyBP/SIP in the process of cardiomyocyte differentiation and heart development. It has also been suggested that elevated levels of CacyBP/SIP may protect cardiomyocytes during myocardial infarction [17]. Research by Kasacka et al. [18] showed an increase in CacyBP/SIP immunoreactivity in rat hearts of the same experimental models of hypertension; however, the intensity of the immunohistochemical reaction showing CacyBP/SIP differed between primary and secondary hypertension. According to the authors’ hypothesis, the differences in protein biosynthesis in the studied types of hypertension may be a consequence of different mechanisms involved in the development of hypertension and organ damage. 

Other studies carried out on various cell lines showed a significantly higher expression of the CacyBP/SIP gene as a result of the induction of oxidative stress [7,19]. This is confirmed by the results of the study by Piotrowska et al., who showed an increase in CacyBP/SIP expression in the hearts of men over 45 years of age, compared younger people. This may be due to an oxidative imbalance resulting from oxidative stress in aging cardiomyocytes [20]. 

Perhaps the lower CacyBP/SIP protein expression found in this study is related to oxidative stress, the role of which in the development of hypertension has not yet been clarified. It is assumed that ROS initially induces the development of hypertension and that the production of reactive oxygen species causes secondary dysfunction of endothelial cells in the course of increasing arterial pressure. More experimental and clinical research is needed to explain the imbalance between free radicals and the antioxidant system that leads to damage to vascular endothelial cells, causing the development of hypertension.

The number of cellular processes in which CacyBP/SIP appears to be involved has increased dramatically after the discovery of various proteins with which it interacts (S100 family proteins, components of the E3 ubiquitin ligase complex, Hsp90 chaperone, cytoskeletal proteins). It has recently been shown that CacyBP/SIP can regulate signaling pathways involving MAP kinases.

The role of ERK1/2 kinase in the pathology of many diseases is widely described in the scientific literature. In the studies conducted so far, the role of ERK1/2 in hypertension was mainly studied in the SHR model [21].

The present study showed a decrease in ERK1/2 immunoreactivity in the cortex and an increase in the adrenal medulla of primary hypertension rats, and vice versa an increase immunoreactivity in the cortex and a decrease in the medulla of DOCA-salt rats. Hypertension is recognized as a chronic inflammatory disease, and mitogen-activated kinases play an important role in the regulation of inflammation. Disturbances in the production of nitric oxide that occur in various pathological conditions, including hypertension, can lead to dysfunction of the adrenal medulla [22]. A study by Vincente et al. [23] showed that increased production of nitric oxide inhibits the secretion of catecholamines in animals. Moreover, nitric oxide can lead to apoptosis of endocrine cells of the adrenal medullary [22]. 

The differences in ERK1/2 immunoreactivity in the adrenal glands of rats with primary and secondary hypertension observed in the present study may be due to various factors disturbing blood pressure homeostasis. The decrease in ERK1/2 immunoreactivity in the adrenal medulla of secondary-hypertensive rats may be due to oxidative stress involving catecholamines. An indicator of oxidative stress is prostaglandin F2a (8-epi-PGF2a). After administration of norepinephrine to rats, an increase in its concentration in the blood was found [24].

The decrease in p-ERK1/2 immunoreactivity demonstrated in the adrenal cortex of SHR rats may indicate disturbed blood pressure homeostasis, which is regulated by hormones produced in the adrenal cortex. Perhaps the factor stimulating the formation of ROS in essential hypertension is angiotensin II, which by increasing the superoxide anion (O_2_^−^) impairs endothelium-dependent vasodilation. Administration of the inhibitor of the enzyme of converting angiotensin to patients with essential hypertension causes a lowering of the concentration of isoprostanes in plasma, which proves the reduction of oxidative stress [25]. 

The observed decrease in the intensity of p-ERK1/2 immunohistochemical staining in the SHR cortex and in medulla of DOCA-salt animals may also be caused by a decrease in kinase activity or an increase in phosphatase levels (perhaps CacyBP/SIP performs this function) and be important in the pathology of primary and secondary hypertension.

Our study showed a significantly higher expression of genes encoding ERK1 and ERK2 in the adrenal glands of rats with both primary and secondary hypertension. The discrepancy between the results of the immunohistochemistry and qRT-PCR is caused by the fact that immunohistochemistry is conducted exclusively on cells of the adrenal glands and qRT-PCR utilizes homogenized adrenal, which also includes other tissue fragments such as blood vessels. 

Activated by environmental stress or inflammatory cytokines, p38 kinase stimulates a cellular biological response appropriate to the signal [14]. It has even been suggested that p38 kinase could be considered a potential therapeutic target for hypertension-related dysfunctions. In the experimental model of primary hypertension, a p38 kinase inhibitor has been shown to cause endothelium-dependent relaxation and lower blood pressure [26,27]. In other studies, p38 inhibitors lowered CRP in ischemic heart disease [28]. A study by Fu et al. showed that the use of a p38 antagonist prevents vascular endothelial dysfunction in rats with primary and secondary hypertension [29].

Assuming that hypertension is a chronic inflammatory disease, organ complications may be associated not only with hemodynamic factors, but also with altered levels of inflammatory markers, e.g., CRP [30]. Experimental studies have shown a reduction in p38 expression in various disease states, in the pathogenesis of which oxidative stress plays a key role [31,32]. In our own research, a significantly higher expression of p38 protein was found in the adrenal glands of hypertensive rats, especially marked in DOCA-salt rats. This may be related to the fact that primary arterial hypertension is a less severe inflammatory disease, as indicated by clinical data [30]. Moreover, the analysis of the localization of p-p38 showed the greatest increase in the immunoreactivity of this kinase in the glomerular layer and medulla of rats with primary hypertension and in the glomerular layer of rats with secondary hypertension, which may suggest a different hormonal function of adrenal cells depending on the etiology of hypertension.

The key role in most signaling pathways is played by the processes of phosphorylation and dephosphorylation, which allow for direct regulation of protein function. These two opposing processes are involved in the stabilization and degradation of proteins, and also affect their localization in the cell. In addition to the numerous confirmed functions of the CacyBP/SIP protein, recent studies indicate a novel phosphatase function of CacyBP/SIP in relation to the MAP kinases. An important function in regulating the phosphatase activity of the CacyBP/SIP protein is played by post-translational modifications, in particular phosphorylation. The CacyBP/SIP protein as a phosphatase of selected MAP kinases has so far been presented in only a few scientific publications [9,33].

So far, there are no data on the cellular location and expression pattern of CacyBP/SIP, ERK1/2 and p38 in hypertension. Since arterial hypertension is accompanied by both abnormal secretion of mineralocorticoids, responsible for the regulation of water and mineral balance, and catecholamines, affecting the functioning of the cardiovascular system, we tracked the localization and expression of the CacyBP/SIP protein and kinases ERK1/2 and p38 in the adrenal cortex and medulla, which have different origins, structures and endocrine functions.

New therapeutic methods are still being sought that would improve the quality of life of patients with hypertension and extend their lifespan. This is achieved through preclinical research on animal models, which have contributed to understanding the pathogenesis of many civilization diseases and establishing effective treatment and prevention. These models are also used to obtain information on the etiopathogenesis of hypertension. Animal models for the study of hypertension can be categorized according to their etiology. SHRs are a good model used to determine the genes responsible for hypertension or to assess organ complications. The etiopathogenesis of SHR has still not been investigated, hence further research is necessary to investigate the molecular mechanisms underlying primary hypertension. Contrastingly, the DOCA-salt model mimics the effects of mineralocorticoid- and glucocorticoid-induced hypertension and is characterized by altered RAAS activity and increased sympathetic activity. It is known that the renin-angiotensin-aldosterone system is involved in the pathogenesis of hypertension by regulating water-electrolyte and acid-base homeostasis. Among the few organs that have the ability to produce prorenin and renin, the adrenal glands stand out. Prorenin and renin, acting on their specific receptors, activate not only the angiotensin pathway, but also the ERK1/2 pathway. The effect of higher expression of the prorenin receptor is an increase in blood pressure and heart rate or aldosteronemia [34]. More and more often, it is proposed to expand the renin-angiotensin-aldosterone system (RAAS) with molecules grouped in three different axes, such as ACE-2/Ang(1-7)/Mas receptor, prorenin/PRR/ERK1/2 kinase and Ang-4/AGTR-4/IRAP [35].

There is a close relationship between the proper functioning of the adrenal glands and hypertension. Aldosterone produced by the glomerular layer of the adrenal glands plays an important role in maintaining blood pressure homeostasis, and one of the most common causes of secondary hypertension is primary hyperaldosteronism. The development of metabolic disorders, including hypertension, may also be the result of the increased secretion of cortisol by the cells of the zona fasciculata or abnormal metabolism of this hormone. Catecholamines released from the adrenal medulla are involved in the neurohormonal regulation of blood pressure, and their association with hypertension is well documented. The secretion of catecholamines is regulated by glucocorticoids. Diversified cell signaling leads to the activation of different mechanisms of receiving and implementing the cell’s response. Mitogen-activated protein kinases (MAPKs) are an important component of intracellular signaling networks. MAPKs are involved in signaling from a wide range of extracellular stimuli, including growth factors, hormones, and cytokines. These kinases are major components of signaling pathways that regulate numerous intracellular events such as proliferation, differentiation, stress response, and gene expression. The MAPK signaling plays an important role in the regulation of steroidogenesis, among others, by influencing, although not entirely clearly, the expression of the StAR, which is responsible for the biosynthesis of steroid hormones in response to an increase in angiotensin II or ACTH. Angiotensin II triggers both the ERK1/2 and p38 cascades, leading to higher expression of StAR and steroid hormones [5]. However, there is evidence indicating that in adrenal cells, activation of the p38 cascade induced by oxidative stress during aging is associated with inhibition of steroidogenesis. The issues described in this study concern phenomena that are potentially important for understanding the mechanisms of development of various forms of hypertension.

In summary, we have provided the first evidence linking CacyBP/SIP to ERK1/2 and p38 kinase in the adrenal gland in experimental models of primary and secondary hypertension. Many of these models have been developed using etiological factors thought to be responsible for human hypertension, such as genetic factors, excessive salt intake, and disorders of the renin-angiotensin-aldosterone system. The results obtained from the conducted research may contribute to a better understanding of the pathophysiological effects of hypertension and the involvement of CacyBP/SIP in connection with MAPK in the mechanisms of development of hypertension disorders.

## 4. Materials and Methods

### 4.1. Experimental Animals

The assumptions, purpose and plan of the study, as well as the approach to animals, were approved by the local Ethics Committee for Studies on Animal Subjects in Białystok.

The experiments were carried out on 24 rats, Wistar males, normotensive and with experimental spontaneous and secondary hypertension. The rats were six weeks of age, and had a body weight of 170–200 g at the beginning of the experiment. The animals were housed at constant humidity (60 ± 5%) and temperature (22 ± 1 °C) and were kept under a 12/12 h light/dark cycle. Rats had free access to standard granulated chow and normal drinking water. 

### 4.2. Experimental Design

The experimental animals were divided into four groups:
ShrSeven rats with genetically determined systemic hypertension, inbred strain established from Wistar rats selected for high blood pressure.Wky Five normotensive Wistar Kyoto rats, being the reference for SHR rats.DOCA-salt Seven Wistar rats, which were uninephrectomized, then rendered hypertensive by high salt diet and deoxycorticosterone acetate (DOCA) injections. Unx Five Wistar rats uninephrectomized only, being the reference for DOCA-salt hypertensive rats.

### 4.3. DOCA–Salt Hypertension

Animals were anesthetized by intraperitoneal injection of pentobarbital sodium (300 μmol or ~70 mg/kg of body weight (b.w.)). The right kidney was removed in all rats through a right lateral abdominal incision. After a one-week recovery period, hypertension was induced for a period of 4 weeks by s.c. DOCA injections (67 μmol or ~25 mg/kg in 0.4 mL/kg of dimethylformamide; DMF) twice weekly and drinking water replaced with 1% NaCl solution. The normotensive control rats were also uninephrectomized but received the vehicle for DOCA (DMF, 0.4 mL/kg, s.c.) twice a week and drank tap water.

### 4.4. Blood Pressure Measurement by an Indirect Method in Wakeful Rats

After a 6-week experiment period, systolic blood pressure (SBP) was measured in all animals using the non-invasive tail cuff method (using a Rat Tail Blood Pressure Monitor, Hugo Sachs Elektronik-Harvard Apparatus, March–Hugstetten, Germany). Blood pressure (BP) measurements were considered valid only if three consecutive readings did not differ by more than 5 mmHg. The average of the three measured values was then recorded. BP measurements showed systolic hypertension in SHR, 2K1C and DOCA-salt rats (those animals had SBP values equal to or higher than 150 mmHg). 

### 4.5. Method of Experimental Material Collection and Fixation

At six weeks of the experiment, adrenal fragments were collected from all rats under deep anesthesia with pentobarbital (50 mg/kg body weight). Obtained adrenal tissues were immediately fixed in 10% buffered formalin and routinely embedded in paraffin or placed in RNA-later solution (AM7024 Thermo Fischer Waltham, MA, USA) and stored in −80 °C.

Adrenal paraffin blocks were cut into 4 µm section and then stained with haematoxylin-eosin for general histological examination and immunohistochemically processed for the detection of CacyBP/SIP, p-ERK1/2 and p-p38. The material stored in the RNA-later solution was subjected to RT-qPCR processing to evaluate the expression of the genes encoding CacyBP/SIP, p-ERK1/2 and p-p38.

### 4.6. Identification of CacyBP/SIP, p-ERK1/2, p-p38 by Immunohistochemical Method

In the immunohistochemical study, the EnVision method was used, as previously described by Kasacka et al. [36]. Immunohistochemistry was performed, using an REAL™ EnVision™ Detection System, Peroxidase/DAB, Rabbit/Mouse detection kit (K5007; Dako Cytomation, Glostrup, Denmark). Immunostaining was performed by the following protocol. Paraffin-embedded sections were deparaffined and hydrated in pure alcohols. For antigen retrieval, the sections were subjected to pre-treatment in a pressure chamber heated for 1 min at 144.7 kPa at 125 °C. During antigen retrieval, sections for detection of CacyBP/SIP, p-ERK1/2 and p-p38 were incubated with Target Retrieval Solution Citrate pH = 6.0 (S 2369 Dako Cytomation, Glostrup, Denmark)). After cooling down to room temperature, the sections were incubated with Peroxidase Blocking Reagent (S 2023 Dako Cytomation) for 5 min to block endogenous peroxidase activity. Subsequently, the sections were incubated with the primary antibody for CacyBP/SIP (rabbit polyclonal antibody to CacyBP/SIP, ab190950 Abcam, Cambridge, UK), p-ERK1/2 (rabbit polyclonal antibody to p-ERK1/2, 44-680G Invitrogen, Waltham, MA, USA) and p-p38 (rabbit polyclonal antibody to p-p38, 44-684G Invitrogen, Waltham, MA, USA). The antisera were previously diluted in Antibody Diluent (S 0809 Dako Cytomation, Glostrup, Denmark) in relation 1:600 for CacyBP/SIP, 1:50 for p-ERK1/2 and 1:50 for p-p38. Incubation with primary antibodies lasted 24 h and was carried out at 4 °C in a humidified chamber. Procedure was followed by incubation (1 h) with secondary antibody (dextran coupled with peroxidise molecules and goat secondary antibody molecules against rabbit and mouse immunoglobulins) (Dako REAL™ EnVision™/HRP Rabbit/Mouse (ENV) K50071 Agilent DGG, Warsaw, Poland). The bound antibodies were visualized by 1 min of incubation with Dako REAL™ DAB+ chromogen. The sections were finally counterstained in hematoxylin QS (H—3404, Vector Laboratories; Burlingame, CA, USA), mounted and evaluated under light microscope. Appropriate washing with Wash Buffer (S 3006 Dako Cytomation, Glostrup, Denmark) was performed between each step (3 times for 5 min). Sections were dehydrated with absolute alcohol followed by xylene, and coverslipped with Entellan (Merck, Darmstadt, Germany). 

Specificity tests, performed for the CacyBP/SIP, p-ERK1/2 and p-p38 antibody included a negative control, where the primary antibodies were omitted, only antibody diluent was used, and a positive control was prepared with specific tissue, as it was recommended by the manufacturer. The positive control for CacyBP/SIP was rat liver, for p-ERK1/2 it was mouse stomach, and for p-p38 it was rat heart. Histological preparations were evaluated using an Olympus BX43 light microscope (Olympus 114 Co., Tokyo, Japan) with an Olympus DP12 digital camera (Olympus 114 Co., Tokyo, Japan) and documented.

### 4.7. RT-qPCR

Samples of adrenal gland were taken from each rat and placed in an RNA-later stabilization solution. Total RNA was isolated using NucleoSpin^®^ RNA Isolation Kit (Machery-Nagel, Oensingen, Switzerland). Quantification and quality control of total RNA was determined using the spectrophotometer—NanoDrop 2000 (ThermoScientific, Waltham, MA, USA). Only RNA samples for which the absorbance ratio at wavelength 260 nm/280 nm was 1.8–2.1 were adopted for the next analysis steps. The mentioned absorbance ratio shows that isolated RNA was not contaminated with protein. An aliquot of 1 μg of total RNA was reverse transcribed into cDNA using iScript^TM^ Advanced cDNA Synthesis Kit for RT-qPCR (BIO-RAD, Barkley, California, USA). Synthesis of cDNA was performed in a final volume of 20 μL using a Thermal Cycler (Model SureCycler 8800, Aligent Technologies, Santa Clara, CA, USA). For reverse transcription, the mixtures were incubated at 46 °C for 20 min, heated to 95 °C for 1 min and finally rapidly cooled at 4 °C.

Quantitative RT-qPCR reactions were performed using Stratagene Mx3005P (Aligent Technologies, Santa Clara, CA, USA) with the SsoAdvanced^TM^ Universal SYBER^®^ Green Supermix (BIO-RAD, Barkley, CA, USA). Specific primers for the CacyBP/SIP (*Cacybp*), ERK1/2 (*Mapk3*, *Mapk1*), p38 (*Mapk14*) and GAPDH (*Gapdh*) were designed by BIO-RAD Company (Barkley, CA, USA). The housekeeping gene GAPDH (*Gapdh)* was used as the reference gene for quantification. In order to determine the amounts of tested genes expression levels, standard curves for each gene were constructed separately with serially diluted PCR products. PCR products were obtained by amplification of cDNA using specific primers as follows: *Cacybp* (qRnoCED0006717, BIO-RAD), *Mapk3* (qRnoCID0002469, BIO-RAD), *Mapk1* (qRnoCID0003206, BIO-RAD), *Mapk14* (qRnoCID0005775, BIO-RAD) and *Gapdh* (qRnoCID0057018, BIO-RAD, Barkley, CA, USA) was carried out in a doublet in a final volume of 20 μL under the following conditions: 2 min polymerase activation at 95 °C, 5 s denaturation at 95 °C, 30 s annealing at 60 °C for 35 cycles. PCR reactions were checked by including no-RT-controls, by omission of templates, and by melting curve to ensure only a single product was amplified. The relative quantification of gene expression was determined by comparison of values of Ct using the ΔΔCt method. All results were normalized to *Gapdh.* To facilitate the analysis of research results, studied proteins and their corresponding gene names are included in Table 3.

### 4.8. Measurement of the Intensity of the Immunohistochemical Reaction

Adrenal sections were made from each animal (three sections each: for immunohistochemistry showing CacyBP/SIP, p-ERK1/2 and p-p38). Five randomly selected microscopic fields (each field 0.785 mm^2^, 200× magnification (20× lens and 10× eyepiece)) from each adrenal section were documented using an Olympus DP12 microscope camera. Each digital image of the adrenal slice was evaluated using Nikon’s NIS Elements Advanced Research microscopy image analysis software, version 3.10. In order to assess the intensity of the immunohistochemical reaction in adrenal cells, the average optical density of the examined objects was measured. The intensity of the immunohistochemical reaction for CacyBP/SIP, p-ERK1/2 and p-p38 was measured in each image and quantified using a grayscale level of 0–256. A value of 0 means a completely white pixel, i.e., minimum light saturation, while 256 means a completely black pixel, i.e., maximum light saturation.

### 4.9. Statistical Analysis

All collected data were statistically analyzed using the Statistica computer package, version 13.3. The corresponding mean values were calculated automatically; due to the lack of normality of the distribution of the obtained results, a non-parametric Kruskal–Wallis test was performed to assess the statistical significance of differences between the groups (WKY/SHR and UNX/DOCA-salt). *p* < 0.05 was taken as the level of significance.

In order to analyze the correlation between the tested proteins, statistically calculated r^2^, the regression equation and the correlation coefficient. The results of this analysis are shown as the β coefficient (this metric represents the percentage of the dependent variable’s change for each unit of the independent variable’s change), r^2^ (shows the percentage of one variable that is responsible for the variability of the other), and the statistical significance (*p*). The relationship between two variables was acknowledged to be statistically significant at the value of the β coefficient for which *p* < 0.05.

## 5. Conclusions

Regulation of specific elements of the signaling pathway involving MAPK and the CacyBP/SIP protein is a very promising therapeutic strategy, especially in relation to the prevention of cardiovascular diseases. Although much remains to be clarified, the results obtained so far, including our research, justify the continuation of this line of research. However, developing new therapeutic strategies targeting the CacyBP/SIP protein, whose new functions are still being discovered, requires a full understanding of its role in both physiological and pathological processes. We are aware of the need for further research using molecular techniques, e.g., microRNA, and more sophisticated quantitative analysis methods in order to better assess the levels and correlations of the tested proteins. We also intend to expand our research to perform co-localization studies using immunofluorescence, which would enable “direct” connection of higher/lower expression of the tested proteins in the same cell. Future research in this area may also focus on further investigation of CacyBP/SIP-mediated signaling pathways and the establishment of selective inhibitors of CacyBP/SIP-mediated signaling to evaluate the prognostic and therapeutic values of hypertension.

## Figures and Tables

**Table 1:** Mean values of systolic blood pressure (mmHg) of rats in control and hypertensive groups ($\bar{x}$  ±  SD).

Rats	Control Group	Hypertensive Group
WKY/SHR	122.3 ± 2.3	160.8 ± 3.3 *
UNX/DOCA-salt	126.0 ± 4.0	180.0 ± 13.0 *

* *p* < 0.05 control group vs. hypertensive group. SHR: spontaneously hypertensive; DOCA-salt: deoxycorticosterone acetate.

**Table 2:** The intensity of immunoreaction determining CacyBP/SIP, p-ERK1/2 and p-p38 in adrenals of normotensive and hypertensive rats (mean ± SE).

		Intensity of Immunohistochemical Reaction in Rat Adrenal Glands Scale from 0 (White Pixel) to 256 (Black Pixel)			
		WKY	SHR	UNX	DOCA-Salt
CacyBP/SIP	zona glomerulosa of adrenal cortex	67.2 ± 3.06	43.5 ± 3.80 *↓	93.8 ± 3.80	75.1 ± 5.76 *↓
	zonae fasciculata and reticularis of adrenal cortex	51.3 ± 1.58	33.9 ± 1.27 *↓	67.5 ± 1.47	53.6 ± 1.89 *↓
	adrenal medulla	83.8 ± 2.82	65.7 ± 3.00 *↓	129.3 ± 5.49	83.9 ± 5.50 *↓
p-ERK1/2	zona glomerulosa of adrenal cortex	145.7 ± 5.01	143.9 ± 4.84 ↓	116.9 ± 3.15	183.4 ± 3.57 *↑
	zonae fasciculata and reticularis of adrenal cortex	84.4 ± 3.30	100.6 ± 3.27 *↑	92.3 ± 3.75	119.6 ± 4.44 *↑
	adrenal medulla	110.5 ± 6.05	169.5 ± 4.78 *↑	197.2 ± 2.86	178.7 ± 4.22 *↓
p-p38	zona glomerulosa of adrenal cortex	84.1 ± 3.93	143.8 ± 6.90 *↑	133.6 ± 4.10	186.4 ± 3.84 *↑
	zonae fasciculata and reticularis of adrenal cortex	64.1 ± 1.92	108.2 ± 4.05 *↑	77.8 ± 3.36	116.0 ± 6.15 *↑
	adrenal medulla	96.7 ± 6.65	148.4 ± 3.62 *↑	86.4 ± 2.41	138.7 ± 3.75 *↑

* *p*  <  0.05 control group vs. hypertensive group; **↑**—intensification of immunohistochemical reaction; **↓**—weakening of immunohistochemical reaction.

**Table 3:** Studied proteins and their corresponding gene names.

Protein Name	Gene Name
CacyBP/SIP	*Cacybp*
ERK1	*Mapk3*
ERK2	*Mapk1*
p38	*Mapk14*

## Data Availability

The datasets generated during and/or analyzed during the current study are available from the corresponding author on reasonable request.

## Supplementary Materials

The following supporting information can be downloaded at: https://www.mdpi.com/article/10.3390/ijms25010084/s1.
