# Supplementary material for: Evaluation of the Expression and Localization of the Multifunctional Protein CacyBP/SIP and Elements of the MAPK Signaling Pathway in the Adrenal Glands of Rats with Primary and Secondary Hypertension
Source: Int J Mol Sci. 2023 Dec 20;25(1):84. doi: 10.3390/ijms25010084 (PMC10779320; doi:10.3390/ijms25010084)
Supplement: Supplementary file 1 [file ijms-25-00084-s001.zip › ijms-2747725-supplementary.pdf]

Table S1. Correlation analysis between the tested proteins in the cortex and medulla of rats with spontaneous hypertension.

SPONTANEOUS HYPERTENSION – ADRENAL CORTEX			
	**CACYBP/SIP**	**ERK1/2**	**P38**
**CACYBP/SIP**	-	β = 0.2001<br>p = 0.2784<br>r^2 = 0.0649	β = 0.0194<br>p = 0.8832<br>r^2 = 0.0012
**ERK1/2**	β = 0.2001<br>p = 0.2784<br>r^2 = 0.0649	-	β = -0.2021<br>p = 0.2181<br>r^2 = 0.0830
**P38**	β = 0.0194<br>p = 0.8832<br>r^2 = 0.0012	β = -0.2021<br>p = 0.2181<br>r^2 = 0.0830	-
**SPONTANEOUS HYPERTENSION – ADRENAL MEDULLA**			
	**CACYBP/SIP**	**ERK1/2**	**P38**
**CACYBP/SIP**	-	β = -0.1431<br>p = 0.1630<br>r^2 = 0.1052	β = -0.0012<br>p = 0.9951<br>r^2 = 0.0000
**ERK1/2**	β = -0.1431<br>p = 0.1630<br>r^2 = 0.1052	-	β = -0.1732<br>p = 0.6986<br>r^2 = 0.0085
**P38**	β = -0.0012<br>p = 0.9951<br>r^2 = 0.0000	β = -0.1732<br>p = 0.6986<br>r^2 = 0.0085	-

The results of the analysis of regression are presented as the **β** coefficient, **r^2**, and the level of statistical significance (**p**, where * $p < 0.05$, † $p < 0.01$, and ‡ $p < 0.001$).

Table S2. Correlation analysis between the tested proteins in the cortex and medulla of rats with secondary hypertension.

SECONDARY HYPERTENSION – ADRENAL CORTEX			
	CACYBP/SIP	ERK1/2	P38
CACYBP/SIP	-	β = 0.2681<br>p = 0.4835<br>r^2 = 0.0277	β = -0.018<br>p = 0.9123<br>r^2 = 0.0007
ERK1/2	β = 0.2681<br>p = 0.4835<br>r^2 = 0.0277	-	β = -0.0109<br>p = 0.9148<br>r^2 = 0.0007
P38	β = -0.018<br>p = 0.9123<br>r^2 = 0.0007	β = -0.0109<br>p = 0.9148<br>r^2 = 0.0007	-
SECONDARY HYPERTENSION – ADRENAL MEDULLA			
	CACYBP/SIP	ERK1/2	P38
CACYBP/SIP	-	β = -0.7261<br>p = 0.2375<br>r^2 = 0.0766	β = -0.5446<br>p = 0.1668<br>r^2 = 0.1034
ERK1/2	β = -0.7261<br>p = 0.2375<br>r^2 = 0.0766	-	β = -0.0316<br>p = 0.8378<br>r^2 = 0.0024
P38	β = -0.5446<br>p = 0.1668<br>r^2 = 0.1034	β = -0.0316<br>p = 0.8378<br>r^2 = 0.0024	-

The results of the analysis of regression are presented as the **β** coefficient, $\mathbf{r^2}$, and the level of statistical significance (**p**, where * $p < 0.05$, † $p < 0.01$, and ‡ $p < 0.001$).
